# Downregulating ANP32A rescues synapse and memory loss via chromatin remodeling in Alzheimer model

**DOI:** 10.1186/s13024-017-0178-8

**Published:** 2017-05-04

**Authors:** Gao-Shang Chai, Qiong Feng, Zhi-Hao Wang, Yu Hu, Dong-Sheng Sun, Xiao-Guang Li, Dan Ke, Hong-Lian Li, Gong-Ping Liu, Jian-Zhi Wang

**Affiliations:** 1 Department of Pathophysiology, School of Basic Medicine, Tongji Medical College, Huazhong University of Science and Technology, Wuhan, 430030 China; 2 the Collaborative Innovation Center for Brain Science, Key Laboratory of Ministry of Education of China for Neurological Disorders, Tongji Medical College, Huazhong University of Science and Technology, Wuhan, 430030 China; 3 Co-innovation Center of Neuroregeneration, Nantong University, Nantong, JS 226001 China

**Keywords:** Alzheimer’s disease, ANP32A, Histone acetylation, Synapse, Memory

## Abstract

**Background:**

The impairment of histone acetylation is causally linked to the cognitive decline in Alzheimer’s disease (AD). In addition to histone acetyltransferases (HATs) and histone deacetylases (HDACs), inhibitor of acetyltransferases (INHAT) can also regulate histone acetylation. As a key component of INHAT, level of ANP32A is selectively upregulated in the brain of AD patients. Here we investigated whether downregulating ANP32A can rescue AD-like synapse and memory deficits.

**Methods:**

RFP-labeled lentiviral ANP32A-shRNA was infused stereotaxically into the hippocampal CA3 region of the human tau transgenic mice (termed htau). The spatial learning and memory were assessed by Morris water maze (MWM). The synaptic function was measured by electrophysiological recording and the spine density was detected by Golgi staining. RT-PCR and Western blotting were used to detect the mRNA and protein levels.

**Results:**

Elevation of ANP32 in htau transgenic mice was correlated with learning deficits, while the hippocampal infusion of lenti-siANP32A to downregulate ANP32A in 12 m-old htau mice could rescue memory loss. Further studies demonstrated that downregulating ANP32A restored synapse morphology and the function. In the brain of htau mice, the acetylated histone decreased while knockdown ANP32A unmasked histone for a robust acetylation with reduced INHAT complex formation. Downregulating of ANP32A also attenuated AD-like tau hyperphosphorylation. Finally, several AD-associated risk factors, including tau accumulation, β-amyloid and H_2_O_2_ exposure, increased ANP32A by activating CCAAT/enhancer binding protein-β (C/EBPβ).

**Conclusion:**

We conclude that downregulating ANP32A rescues synaptic plasticity and memory ability by reducing INHAT formation and unmasking histone for hyperacetylation. Our findings reveal novel mechanisms for AD memory loss and potential molecular markers for protection.

**Electronic supplementary material:**

The online version of this article (doi:10.1186/s13024-017-0178-8) contains supplementary material, which is available to authorized users.

## Background

Synapse impairment and memory deficit are hallmark pathology and symptom of Alzheimer’s disease (AD), while the underlying mechanisms are currently unclear. Recent studies suggest that epigenetic modifications, particularly histone acetylation in the nervous system, play a critical role in regulating gene expression for long-term memory [1]. The chromatin remodeling *via* histone acetylation is a key mechanism to control gene expression [2]. Histone acetylation diminishes the electrostatic affinity between neighboring histones and the DNA, and consequently promotes a more open chromatin structure that allows for transcription of the memory-related genes [3].

Acetylation and deacetylation of histone is catalyzed by histone acetyltransferases (HATs) and histone deacetylases (HDACs), respectively. Reduced histone acetylation has been observed in animal models of neurodegenerative diseases including AD, which are characterized by cognitive decline [4]. So far, most studies aimed at pharmacologically increasing histone acetylation for therapeutic purpose mainly focused on approaches manipulating activity of HATs or HDACs. However, histone acetylation is also suppressed by a cellular complex termed inhibitor of acetyltransferases (INHAT) through histone-masking, in which INHAT binds to histones and masks their access to HAT [5, 6].

As key components of INHAT, the levels of ANP32A (also termed as I_1_^PP2A^) and SET (also termed as I_2_^PP2A^) are selectively upregulated in the brain regions affected with neurofibrillary pathology in AD [7–10]. Both ANP32A and SET are endogenous PP2A inhibitors, so that a causal association of ANP32A or SET with tau hyperphosphorylation has been reported [7, 8, 11]. However, whether and how ANP32A plays a role in the memory impairment of AD has not been reported.

In the present study, we found that downregulating C/EBPβ-associated elevation of ANP32A rescues synaptic plasticity and cognitive functions by unmasking histone for a robust acetylation in a well-recognized mouse model of AD.

## Methods

### Materials, cell culture and PP2A activity assay

All the antibodies used in this study are listed in Additional file 1: Table S1. Aβ peptide preparation, cell culture and PP2A activity assay, and the plasmids used in the present study please see Additional file 2. 

### Animals

Human tau transgenic mice (STOCK Mapttm1 (EGFP) Klt Tg (MAPT)8cPdav/J) were purchased from Jackson lab. All animal experiments were performed according to the ‘Policies on the Use of Animals and Humans in Neuroscience Research’ revised and approved by the Society for Neuroscience in 1995, and the animal study was approved by the Academic Review Board of Tongji Medical College, Huazhong University of Science and Technology.

### Production and brain infusion of lentiviral constructs

The ANP32A-shRNA sequence is CGGATTTATTTAGAGCTGC, and it was cloned into LentiLox 3.7. The RFP sequence was driven by a cytomegalovirus (CMV) promoter and terminated using polyadenylation signal in the 3’ long terminal repeat (LTR). The 3rd generation packaging systems was used for lentiviral production. These vectors include: pMDLg/pRRE (gag/pol elements), pRSV-REV, and pMD.G (env elements). The RFP-labeled lentiviral ANP32A-shRNA (LV-siANP) and the scrambled control (LV-siC) were constructed according to the standard procedure. The recombinant lentivirus was produced by transient transfection of HEK293T cells using the calcium phosphate method; the virus was harvested at 48 h and 72 h posttransfection and purified by centrifugation at 4 °C. The titer of the virus was 2×10^9^ TU/ml. The in vivo knockdown efficiency was measured by Western blotting and RT-PCR after injection of the virus into the hippocampal CA3 of mice brains.

For brain injections, male 11 m-old htau mice, or 11 m-old wild-type littermates, were positioned respectively in a stereotaxic instrument, then 2 μl LV-siANP32A (htau-siANP), LV-siC (htau-siC) were bilaterally injected into the hippocampal CA3 region (AP ±2.0, ML -1.5, DV -2.0) at a rate of 0.50 μl/min. The needle was left in place for ~3 min before being withdrawn. All mice were kept at 24 ± 2 °C on daily 12 h light-dark cycles with ad libitum access to food and water. The injection did not significantly increase the death rate or change the normal activity of the mice compared with the non-injected controls. The hippocampal CA3 region was used for the biochemical measurements.

### Behavioral tests

Four weeks after brain infusion of the viral vectors, the spatial learning and memory were assessed by Morris water maze (MWM) test [12, 13]. For spatial learning, mice were trained in water maze to find a hidden platform for 6 consecutive days, 4 trials per day with a 30 s interval from 14:00 to 20:00 pm. On each trial, the mice started from one of the four quadrants facing the wall of the pool and ended when the animal climbed on the platform. If the mice did not locate the platform within 60 s, they were guided to the platform. The swimming path and the time used to find the platform (latency) or pass through the previous platform quadrant were recorded each day by a video camera fixed to the ceiling, 1.5 m from the water surface. The camera was connected to a digital-tracking device attached to an IBM computer. The spatial memory was tested 1 day after the last training. The longer a mouse stayed in the previous platform-located quadrant, the better it scored the spatial memory.

### Real time PCR

Total RNA (3 μg in 25 μl), isolated using TrizolTM (Invitrogen, CA), was reversely transcribed and the produced cDNA (1 μl) was used for real time PCR with primer sets: 5’-CAAACAATACCGAAGGGCACAG-3’ and 5’-AAGAGGGCTAGATAATCAGAAGACAGA-3’ for synaptophysin, 5’- CTTTGCTTGTTTATTTTGCTTC-3’ and 5’-CCAATGTGTTTATCTGTGACTG-3’ for synapsin I, 5’-TATGCTCTTTGGGTCAGTCTCGTT-3’ and 5’- GTCCCTTTATCCTCCGTCTTTCTT-3’ for NR2B, 5’- TCAAGGAAAGCAGAAGGGGAAA-3’ and 5’-TGTGGAATGGAATGATAGGCGA-3’ for NR2A, 5’-CACATGTAGCCGGAGTGATG-3’ and, 5’-CACTCAAGAGGATGGGGAAA-3’ for GluR1, 5’-ATTTCGGGTAGGGATGGTTC-3’ and 5’-ACCATCCTTCACTGGCATTC-3’ for GluR2, 5’-CACGTTTTCTCGGTAGGCATT-3’ and 5’- AGGGGACCTGGAAGTATTGGC-3’ for ANP32A, and 5’-AGCCTTCCTTCTTGGGTAT-3’ and 5’-GCTCAGTAACAGTCCGCCTA-3’ for β-actin.

### Golgi impregnation and dendritic morphology analysis

For Golgi stain, the mice were anesthetized and then perfused transcardially with 4% paraformaldehyde, and brain tissue was processed as described [14]. Individual sections were incubated overnight at room temperature in water solution of 3.5% K_2_Cr_2_O_7_ and 0.4% OsO_4_. The sections were then sandwiched in two glass slides and incubated in 1% AgNO_3(aq)_ for 5 h at room temperature in dark. Then the slide assemblies were dismantled in water and the sections were mounted on gel-coated slides (0.5% porcine gelatin), dehydrated in a series of graded ethanol rinses, cleared with Histoclear, and cover slipped with cytoseal. The images were taken using Olympus BX60 (Tokyo).

The spine density was determined in the segments of dendrites at a distance of 190-210 μm (distal) from the soma. To acquire images for spine analysis, the dendritic segments were imaged under bright-field illumination on a Zeiss Axioimager microscope with a 63× oil immersion objective, and spine morphology was analyzed according to a previously reported method [15], which does not assess spine density in a 3 dimensional manner but focuses on spines paralleled to the plane of section. Although the method may underestimate the total number of spines, it facilitates a direct comparison of treatment groups when they are analyzed in an identical manner. Image J software was used to calculate linear spine density [16], which was presented as the number of spines per 10 μm of dendrite length. On the basis of morphology, spines were classified into thin (spines with a long neck and a visible small head) and mushroom (big spines with a well-defined neck and a very voluminous head) spines. Data from 5-7 neurons were averaged per animal and used in further statistical analysis.

### Electrophysiological analysis

Mice brains were cut into horizontal sections of 400 μm thickness by a vibration microtome in cold artificial cerebrospinal fluid (aCSF) containing (in mM) NaCl 126, KCl 2.5, NaHCO_3_ 26, NaH_2_PO_4_ 1.25, CaCl_2_ 2, MgCl_2_ 2, glucose 10, equilibrated with 95% O_2_ and 5% CO_2_. Then the hippocampal slices were transferred into oxygen-enriched artificial cerebrospinal fluid (aCSF) to recover for 30 min. Individual slices were laid down over an 8×8 array of planar microelectrodes, each 50×50 mm in size, with an interpolar distance of 450 mm (MED-P5455; Alpha MED Sciences, Kadoma, Japan) and kept submerged in aCSF (4 ml/min; 30 °C) with a nylon mesh glued to a platinum ring. Voltage signals were acquired using the MED64 System (Alpha MED Sciences). Field excitatory postsynaptic potentials (fEPSPs) were recorded from CA3 in hippocampus by stimulating mossy fibers. Stimulation intensity was adjusted to evoke fEPSP amplitudes that were 30 % of maximal size. Long-term potentiation was induced by applying 1 train of high-frequency stimulation (100 Hz, 1 second duration at test strength). Paired-pulse facilitation (PPF) was examined by applying pairs of pulses, which were separated by 50–300 ms intervals.

### Western blotting

Western blotting was performed as described previously [17]. Mice were decapitated after the spatial memory retention test. The hippocampal CA3 region (where virus infected), cortex and cerebellum were rapidly removed and homogenized at 4 °C using a Teflon glass homogenizer with 50 mM Tris-HCl (pH 7.4), 150 mM NaCl, 10 mM NaF, 1 mM Na_3_VO_4_, 5 mM EDTA, 2 mM benzamidine, and 1 mM phenylmethylsulfonyl fluoride. The extract was mixed with sample buffer (3:1, v/v) containing 200 mM Tris-HCl (pH 7.6), 8% SDS, 40% glycerol, 40 mM dithiothreitol, then boiled for 10 min. The proteins were separated by 10% SDS-polyacrylamide gel electrophoresis and transferred to nitrocellulose membranes. The membranes were blocked with 5% nonfat milk dissolved in TBSTween-20 (50 mM Tris-HCl (pH 7.6), 150 mM NaCl, 0.2% Tween-20) for 1 h and probed with primary antibody at 4 °C overnight. Finally, the blots were incubated with anti-rabbit or anti-mouse IgG conjugated to IRDyeTM (800CW) for 1 h at 15-25 °C and visualized using the Odyssey Infrared Imaging System (Licor biosciences, Lincoln, NE, USA).

### Immunohistochemistry

In brief, mice were sacrificed and perfused through aorta with 100 ml 0.9 % NaCl followed by 400 ml phosphate buffer containing 4 % paraformaldehyde. Brains were removed and postfixed in perfusate overnight and then cut into sections (20 μm) with a vibratome (Leica, Nussloch, Germany; S100, TPI). Free floating sections were blocked with 3% H_2_O_2_ in anhydrous methanol for 30 min and nonspecific sites were blocked with bovine serum albumin (BSA) for 30 min at room temperature. Sections were then incubated overnight at 4 °C with primary antibodies. Immunoreaction was developed using HistostainTM-SP kits and visualized with diaminobenzidine (brown color). For each primary antibody, 3-5 consecutive sections from each brain were used. The images were observed using a microscope (Olympus BX60, Tokyo, Japan).

### Immunoprecipitation

The protein extracts from mouse hippocampal CA3 region injected with virus were subsequently incubated for overnight at 4 °C with the precipitating antibodies. Twenty microliters of a 1:1 suspension of protein G-Sepharose beads was added to the mixture and incubated for 2 h at 4 °C with gentle rotation. The beads were collected by centrifugation and washed extensively with cell lysis buffer. The bound proteins were dissociated by boiling the beads in 2×Laemmli sample buffer and separated by SDS-polyacrylamide gel electrophoresis (SDS-PAGE).

### Statistical analysis

The data was expressed as mean±SD or mean±SEM (for animal behavior measurement) and statistical comparisons were performed using SPSS 12.0 statistical software (SPSS Inc., Chicago, Illinois). Student’s unpaired t-test, and one-way analysis of variance procedure followed by Bonferroni’ s post hoc test, or two-way repeated measures ANOVA and Bonferroni’ s post hoc test were used to determine the statistical significance of means.

## Results

### ANP32A elevation is correlated with learning deficit in htau mice

**Fig. 1:**
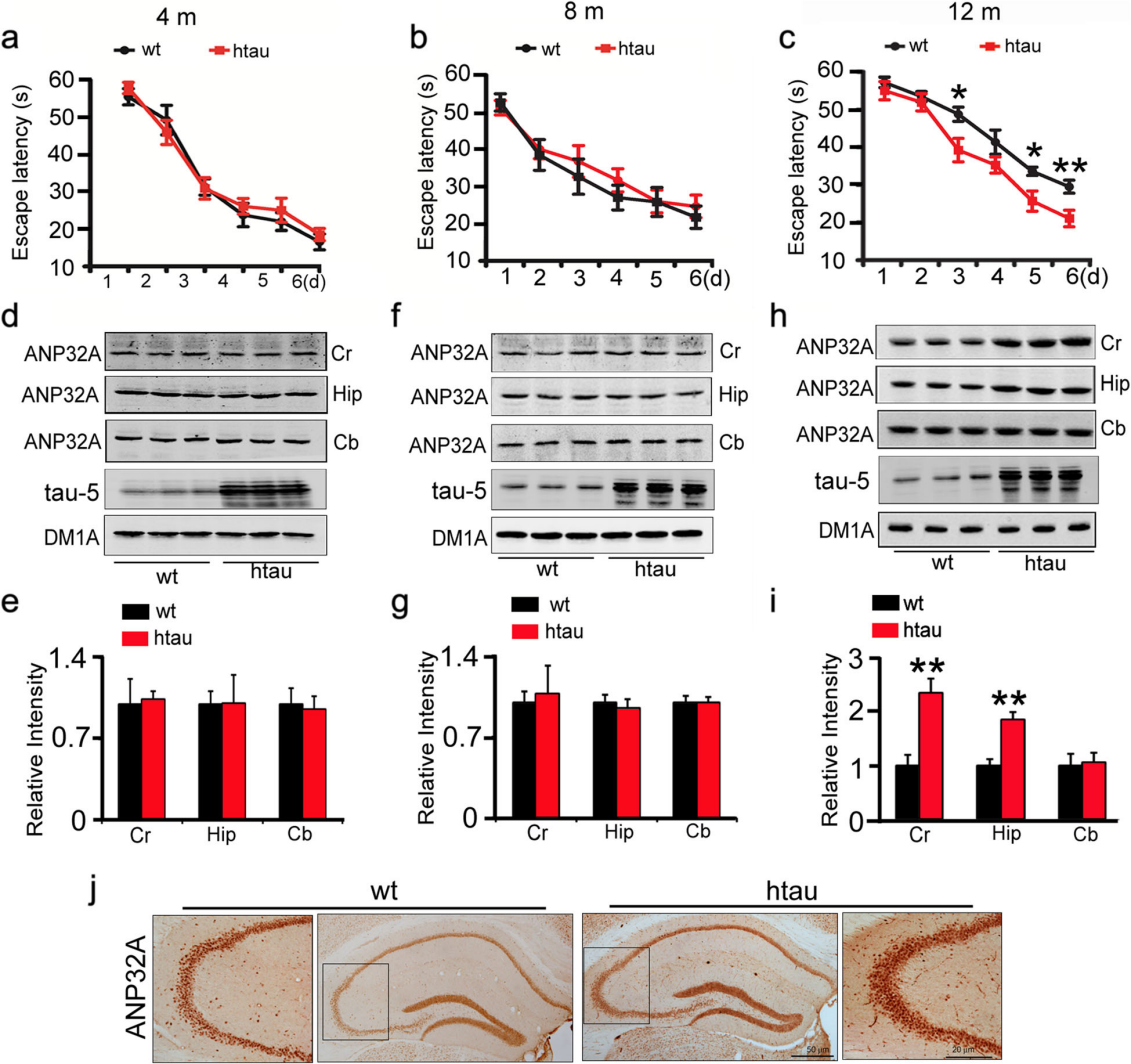
ANP32A elevation is correlated with learning deficit in htau mice. **a**–**c** Only the 12 m- but not 4 m- and 8 m-old htau transgenic mice show increased latency during 6 days learning trials in MWM test when compared with the age-matched wild-type (wt) littermates (a: wt, *n*=10, htau, *n*=9; b: wt, *n*=15, htau, *n*=12; c: *n*=9 each group). Data were presented as mean ± s.e.m. *, *p*<0.05, **, *p*<0.01 *vs* wt (one-way repeated measures ANOVA and Bonferroni’ s post hoc test). **d**–**i** Only the 12 m- but not 4 m- and 8 m-old htau mice show increased ANP32A level in cortex (Cr) and hippocampus (Hip) compared with the wt littermates, and no difference of ANP32A was detected in cerebellum (Cb) measured by Western blotting. Tubulin probed by DM1A was used as a loading control. Data were presented as mean ± SD. **, *p*<0.01 *vs* wt (Unpaired Student's *t*-test (two-tailed)). **j** The representative images of ANP32A in hippocampus measured by immunohistochemical staining (*n*=3-6 slices from 3 mice for each group)

ANP32A is significantly increased in the AD brains [7, 8], but the role of ANP32A in learning and memory is not known. We observed by Morris water maze (MWM) test that the human tau transgenic mice (htau) show learning deficits at 12 m but not at 4 m and 8 m-old compared with the age-matched wild-type littermates (Fig. 1a–c). Interestingly, the htau mice show significant elevation of ANP32A only at 12 m but not at 4 m and 8 m-old, and the increase was only detected in the hippocampus and cortex but not in cerebellum (Fig. 1d–i), the latter is not directly involved in learning and memory. A remarkable increase of ANP32A protein level was also detected by immunohistochemical staining in hippocampal CA3 of 12 m-old htau mice (Fig. 1j). These data indicate that ANP32A may play a role in learning performance.

### Downregulating ANP32A rescues memory in htau mice

**Fig. 2:**
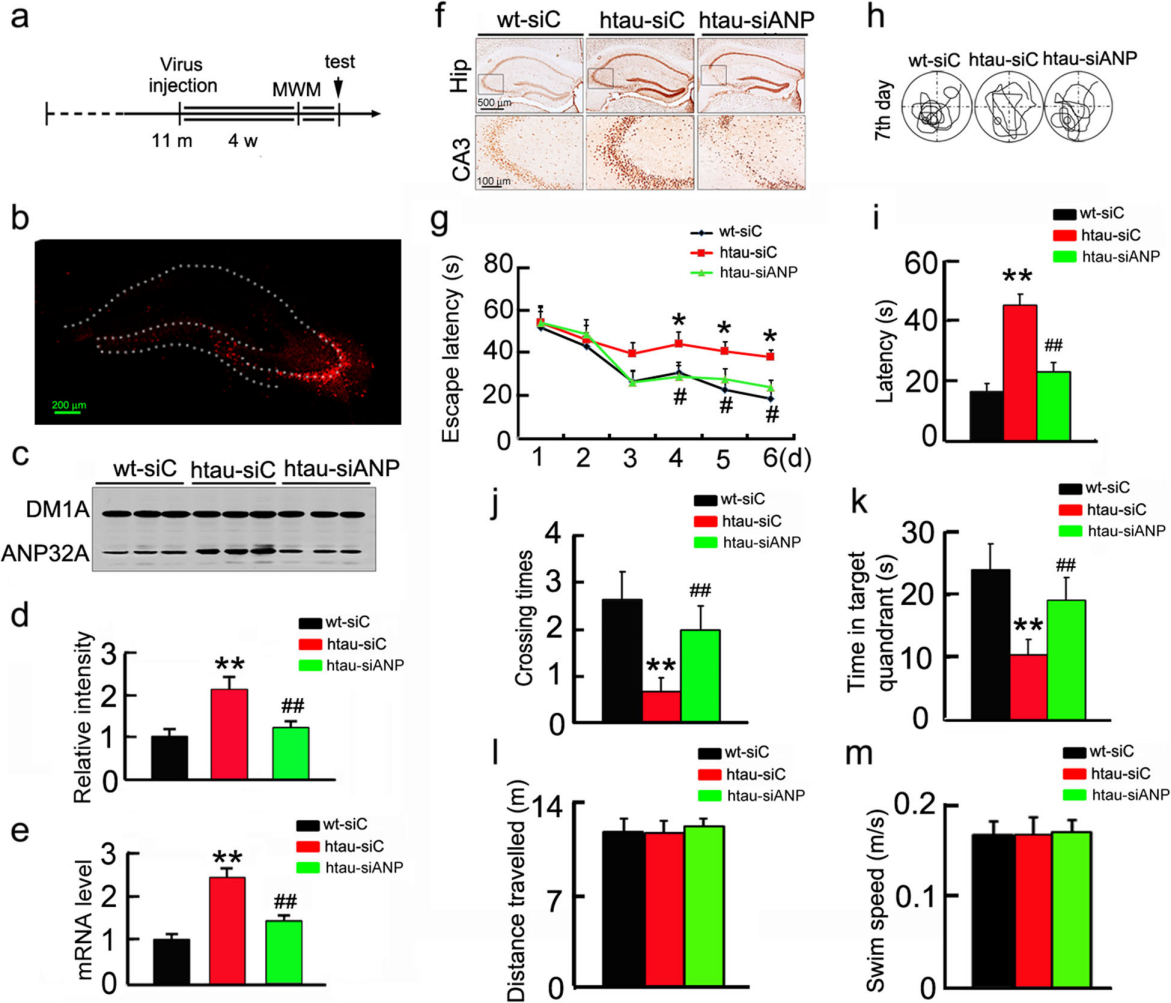
Downregulating ANP32A improves spatial learning and memory in htau mice. **a** Schematics of the procedure. Lentivirus co-expressing RFP and ANP32A shRNA (siANP) or the scrambled control shRNA (siC) (2×10^9^ TU/ml) were stereotaxically injected into the hippocampal CA3 of 11 m-old htau mice (htau-siC and htau-siANP) or the age-matched wild-type littermates (wt-siC). One month later, learning and memory were detected in Morris water maze (MWM) and then the mice were sacrificed for the biochemical measurements. **b** The representative image confirming expression of the injected lentivirus in hippocampal CA3. **c**, **d** The knockdown efficiency of ANP32A protein in hippocampal CA3 measured by Western blotting and quantitative analysis. Data were presented as mean ± SD (two-way ANOVA followed by Bonferroni’ s post hoc test). **e** The knockdown efficiency of ANP32 mRNA in the hippocampal CA3 detected by RT-PCR. Data were presented as mean ± SD (two-way ANOVA followed by Bonferroni’ s post hoc test). **f** The knockdown efficiency of ANP32A protein in hippocampal CA3 measured by immunohistochemistry. **g** Arresting ANP32A overexpression improves spatial learning shown by the reduced escape latency in htau mice measured in MWM. Data were presented as mean ± s.e.m (two-way repeated measures ANOVA followed by Bonferroni’ s post hoc test). **h** The representative swimming traces of the mice recorded in the maze after removed the platform in 7th day. **i**–**k** Arresting ANP32A overexpression improves spatial memory shown by the reduced latency to reach the platform quadrant (i), the increased crossing times in the platform site (j) and time spent in the target quadrant (k) measured at day 7 by removed the platform (*n* = 11 each group). Data were presented as mean ± SD (two-way ANOVA followed by Bonferroni’ s post hoc test). **l**, **m** Arresting ANP32A overexpression did not change the distance travelled (l) and swimming speed (m) of the mice in water maze task (two-way ANOVA followed by Bonferroni’ s post hoc test). *, *p*<0.05; **, *p*<0.01 *vs* wt-siC; ^#^, *p*<0.05; ^##^, *p*<0.01 *vs* htau-siC

To test whether knockdown ANP32A could rescue memory deficits, we constructed lentivirus co-expressing ANP32A shRNA (LV-siANP) or the scrambled control shRNA (LV-siC), and injected the virus into the hippocampal CA3 of 11 m-old htau transgenic mice (Fig. 2a). Four weeks after the injection, a robust virus transfection was seen by direct fluorescent imaging (Fig. 2b), and the knockdown efficiency was confirmed by the significantly reduced protein and mRNA levels of ANP32A in hippocampal CA3 of htau mice measured by Western blotting (Fig. 2c and d), RT-PCR (Fig. 2e) and immunohistochemical staining (Fig. 2f). With ANP32A knockdown, the improved learning ability was shown by the reduced escape latency during 6 days training trials (Fig. 2g); and a remarkably improved memory was also detected at day 7 by removed the platform, demonstrated by the decreased latency to reach the target quadrant, the increased time crossings and time spent in the target quadrant (Fig. 2h–k). Swimming behavior was similar among the groups (Fig. 2l and m). These data together demonstrate that arresting ANP32A elevation in hippocampal CA3 can rescue learning and memory decline in htau transgenic mice.

### Downregulating ANP32A preserves synaptic morphology and the functions

**Fig. 3:**
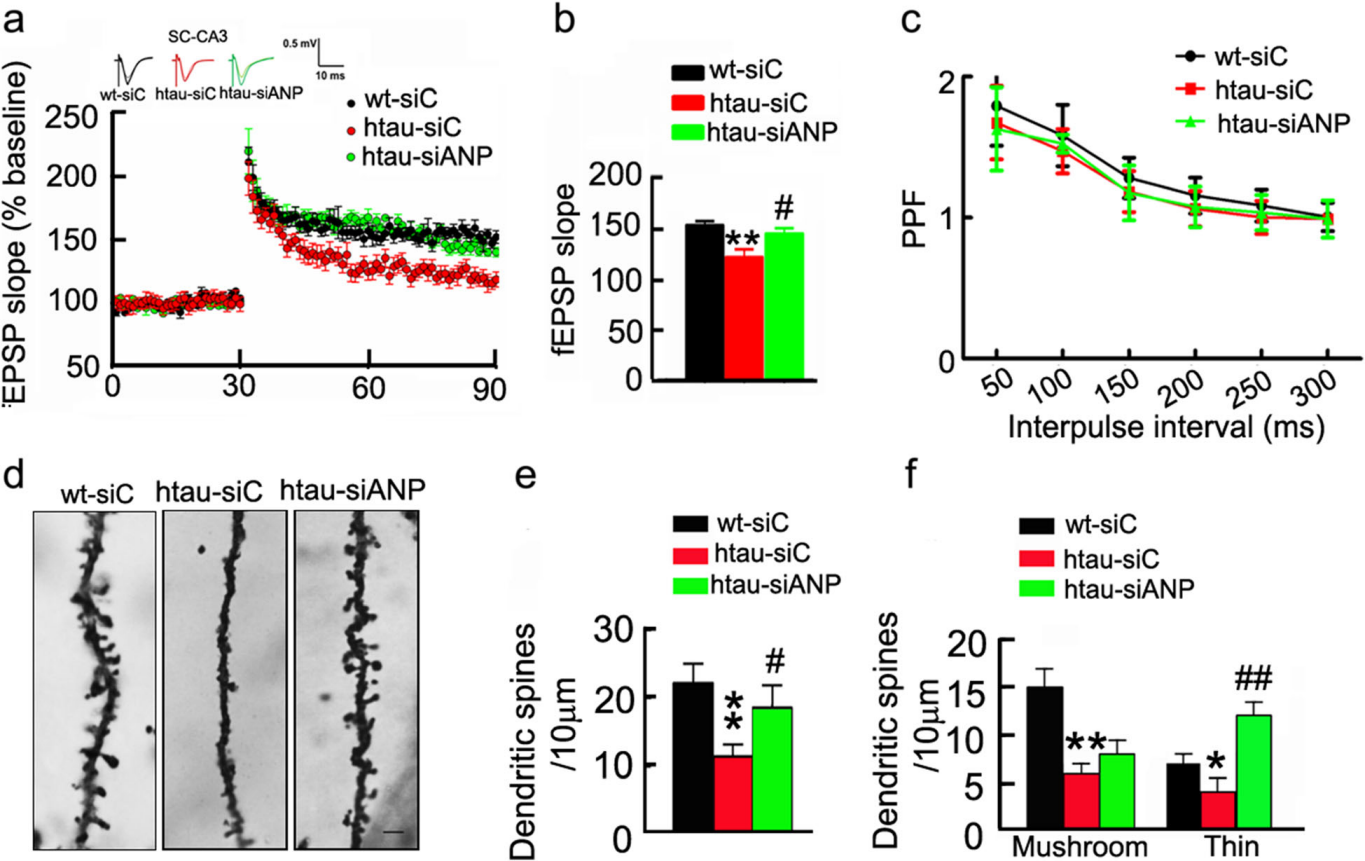
Downregulating ANP32A improves synaptic plasticity. **a**–**c** Arresting ANP32A overexpression in htau mice restores the slopes of fEPSP (a,b) with no influence on paired-pulse ratio (c) in hippocampal CA3 by stimulating mossy fibers. (*n*=7-8 slices from 4 mice for each group). **d**–**f** Arresting ANP32A overexpression in htau mice restores total, thin- and mushroom-shaped spines in the hippocampal CA3 neurons (at least 20 neurons from six to seven mice per group were analyzed by the Sholl). Bar=5 μm. Data were presented as mean ± SD. *, *p*<0.05*,* **, *p*<0.01 *vs* wt-siC; ^#^, *p*<0.05; ^##^, *p*<0.01 *vs* htau-siC (two-way ANOVA followed by Bonferroni’ s post hoc test)

To explore the underlying mechanisms of knockdown ANP32A improving cognitive functions, we first measured the synaptic transmission by electrophysiological recording in the acute brain slices. We observed that the slope of fEPSP in 12 m-old htau mice was decreased compared with the age-matched littermates, while downregulation of ANP32A by LV-siANP32A significantly increased fEPSP slope when compared with the LV-C control mice (Fig. 3a and b). Using a paired-pulse protocol to determine the paired-pulse facilitation (PPF) of the fEPSP at mossy fiber-CA3 circuit, we did not find significant difference among three groups (Fig. 3c), indicating knockdown ANP32 did not significantly affect the presynaptic functions.

The spine generation and mushroom spine formation play a crucial role in learning and memory. Significant reduction of mushroom and thin-shaped spines was also detected in hippocampus of 12 m-old htau transgenic mice, while knockdown of ANP32A restored the spine density (Fig. 3d–f).

**Fig. 4:**
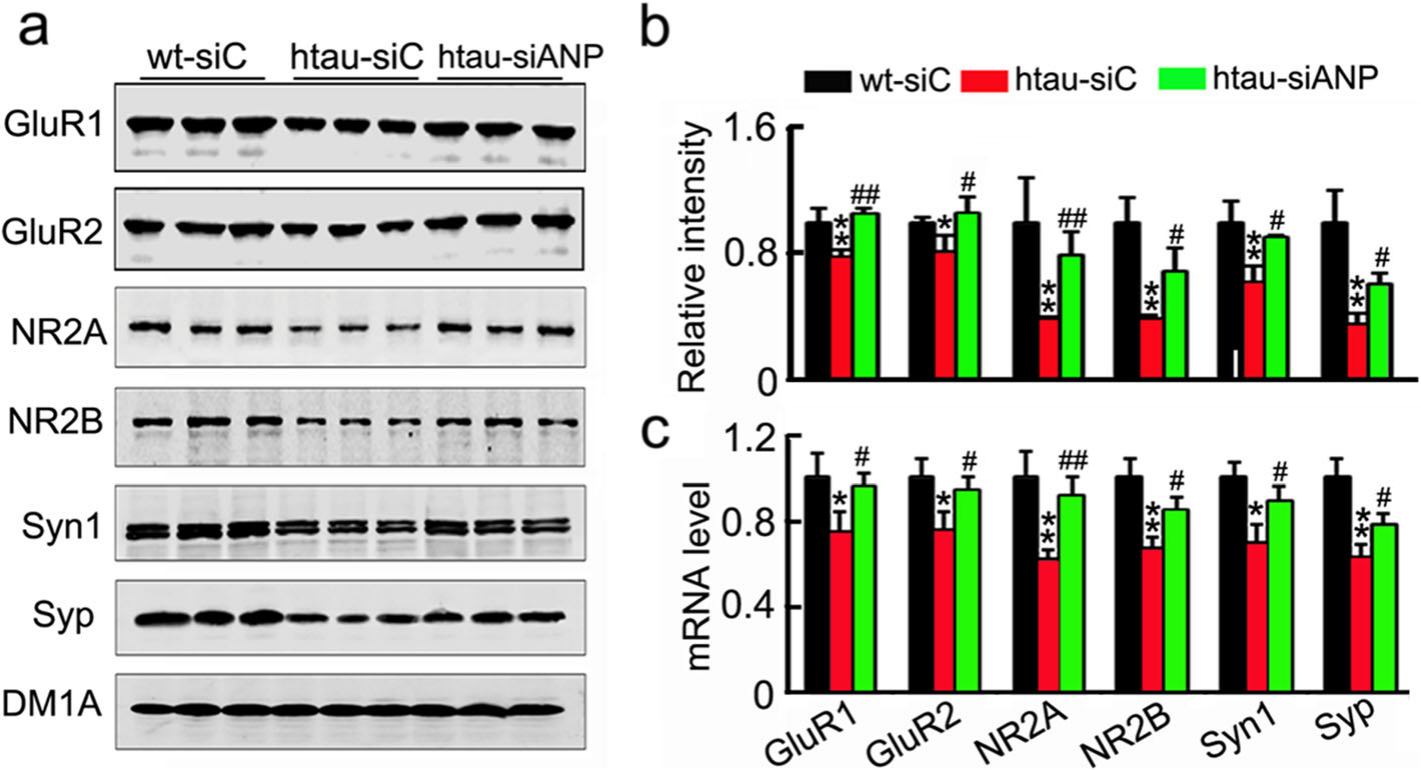
Downregulating ANP32A increases expression of synapse-related proteins. **a**, **b** Arresting ANP32A overexpression in htau mice increases the level of several synaptic proteins in hippocampal CA3, detected by Western blotting. **c** Arresting ANP32A overexpression in htau mice increases mRNA level of several synaptic proteins in the hippocampal CA3, detected by RT-PCR. Data were presented as mean ± SD. *, *p*<0.05, **, *p*<0.01 *vs* wt-siC; ^#^, *p*<0.05; ^##^, *p*<0.01 *vs* htau-siC (two-way ANOVA followed by Bonferroni’ s post hoc test)

To verify the molecular mechanisms underlying the altered synapse morphology, we measured the levels of synapse-associated proteins. We observed that the protein (Fig. 4a and b) and mRNA (Fig. 4c) levels of presynaptic proteins including synaptophysin (Syp) and synapsin I (Syn1) and postsynaptic proteins including N-methyl-D-aspartate receptor type 2A (NR2A) and N-methyl-D-aspartate receptor type 2B (NR2B), AMPA receptor subunits GluR1 and GluR2 were decreased in 12 m-old htau mice compared with the age-matched control mice, while knockdown ANP32A with LV-siANP32A restored the protein and mRNA levels of these synaptic proteins in htau mice to the control levels. These data together suggest that downregulating ANP32A efficiently rescues the synapse impairments in htau transgenic mice.

### Knockdown ANP32A restores histone acetylation by blocking INHAT formation

**Fig. 5:**
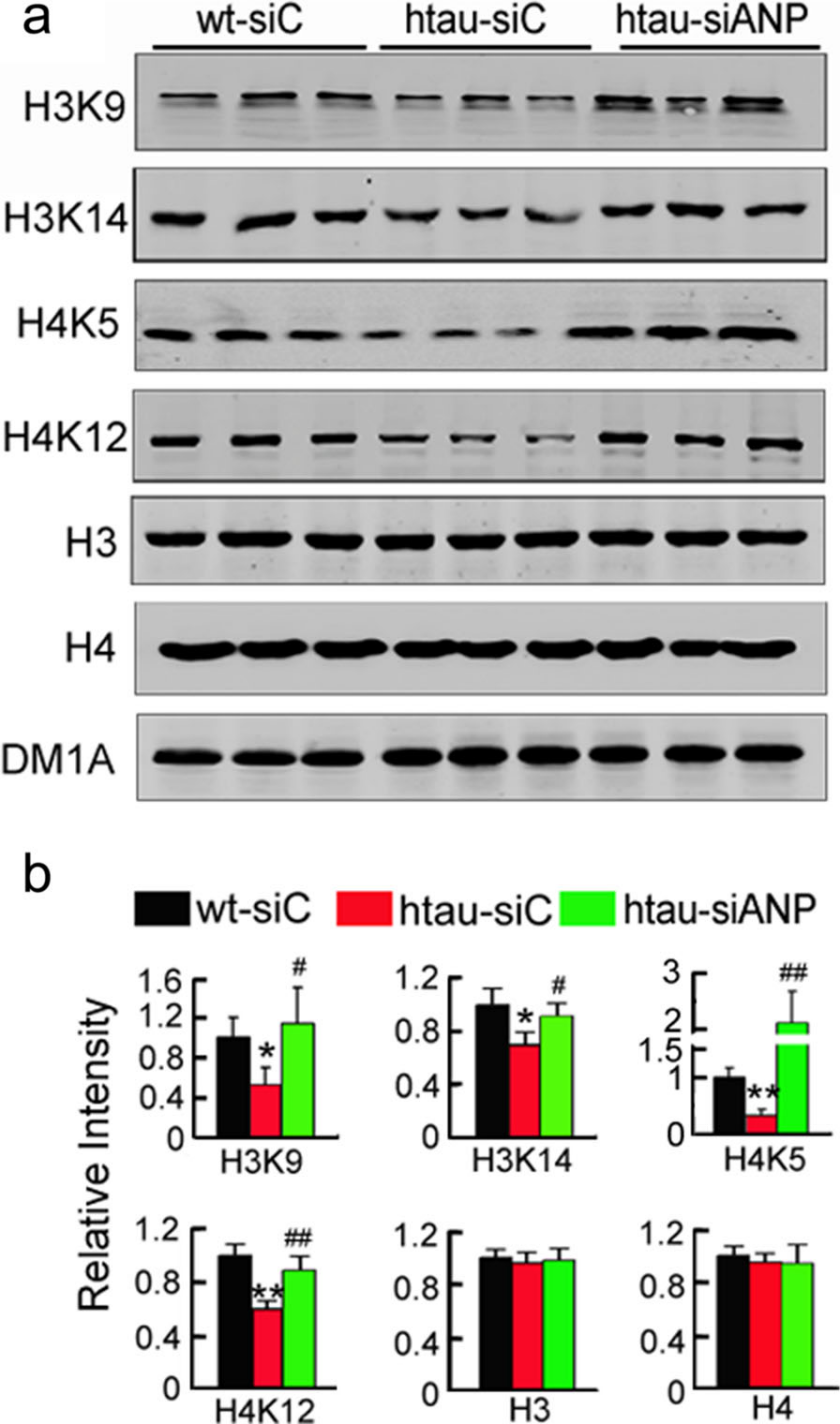
Downregulating ANP32A increases histone acetylation. **a**, **b** Arresting ANP32A overexpression in htau mice increases levels of the acetylated histone in the hippocampal CA3 subset, measured by Western blotting. Data were presented as mean ± SD. *, *p*<0.05, **, *p*<0.01 *vs* wt-siC; ^#^, *p*<0.05; ^##^, *p*<0.01 *vs* htau-siC (two-way ANOVA followed by Bonferroni’ s post hoc test)

Histone acetylation regulates expression of synaptic proteins [18]. The acetylation of H3 at K9 and K14 (H3K9, H3K14) and H4 at K5 and K12 (H4K5, H4K12) were significantly decreased in 12 m-old htau mice compared with the age-matched littermates, while knockdown ANP32A restored the acetylated levels of H3 and H4 without changing the total level of H3 and H4 subunits (Fig. 5).

**Fig. 6:**
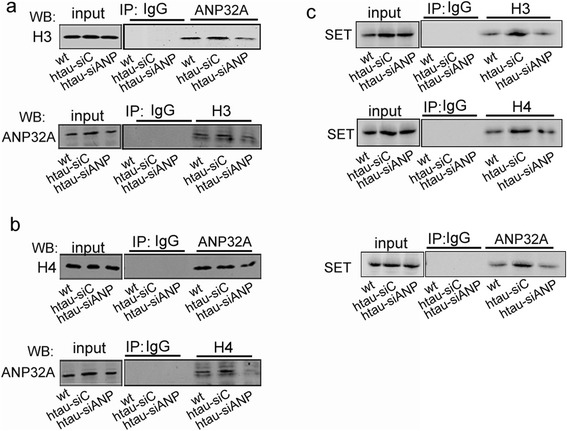
Downregulating ANP32A decreases formation of INHAT complex. **a** The association level between ANP32A and histone 3 (H3) increased in hippocampal CA3 of htau mice, while siANP32A reduced the interaction detected by co-immunoprecipitation using antibodies as indicated on the blots. **b** The association level between ANP32A and histone 4 (H4) increased in hippocampal CA3 of htau mice, while siANP32A reduced the interaction detected by co-immunoprecipitation. **c** The association levels between SET and H3 or H4 or ANP32A increased in hippocampal CA3 of htau mice, while siANP32A reduced the interaction detected by co-immunoprecipitation

ANP32A and SET can bind histone to form an inhibitory complex (i.e., INHAT) that masks the histone acetylation by HAT [5]. To verify the mechanisms underlying the inhibited histone acetylation by ANP32A, we first examined the interaction of ANP32A with histone by immunoprecipitation assay. An increased association of H3 or H4 with ANP32A was observed in htau mice compared with the control mice, while siANP32A reduced the interaction of ANP32A with H3 or H4 (Fig. 6a and b). Knockdown ANP32A also decreased interaction of ANP32A, H3 and H4 with SET, another subunit of INHAT with no significant change of SET protein level (Fig. 6c, lane 2 and 3 in the Input). These data suggest that ANP32A inhibits histone acetylation by direct interaction, and knockdown ANP32A reduces association of ANP32A with SET to form the inhibitory INHAT complex and thus unmasks the histone for a robust acetylation.

### AD-risk factors increase ANP32A by stimulating C/EBPβ

**Fig. 7:**
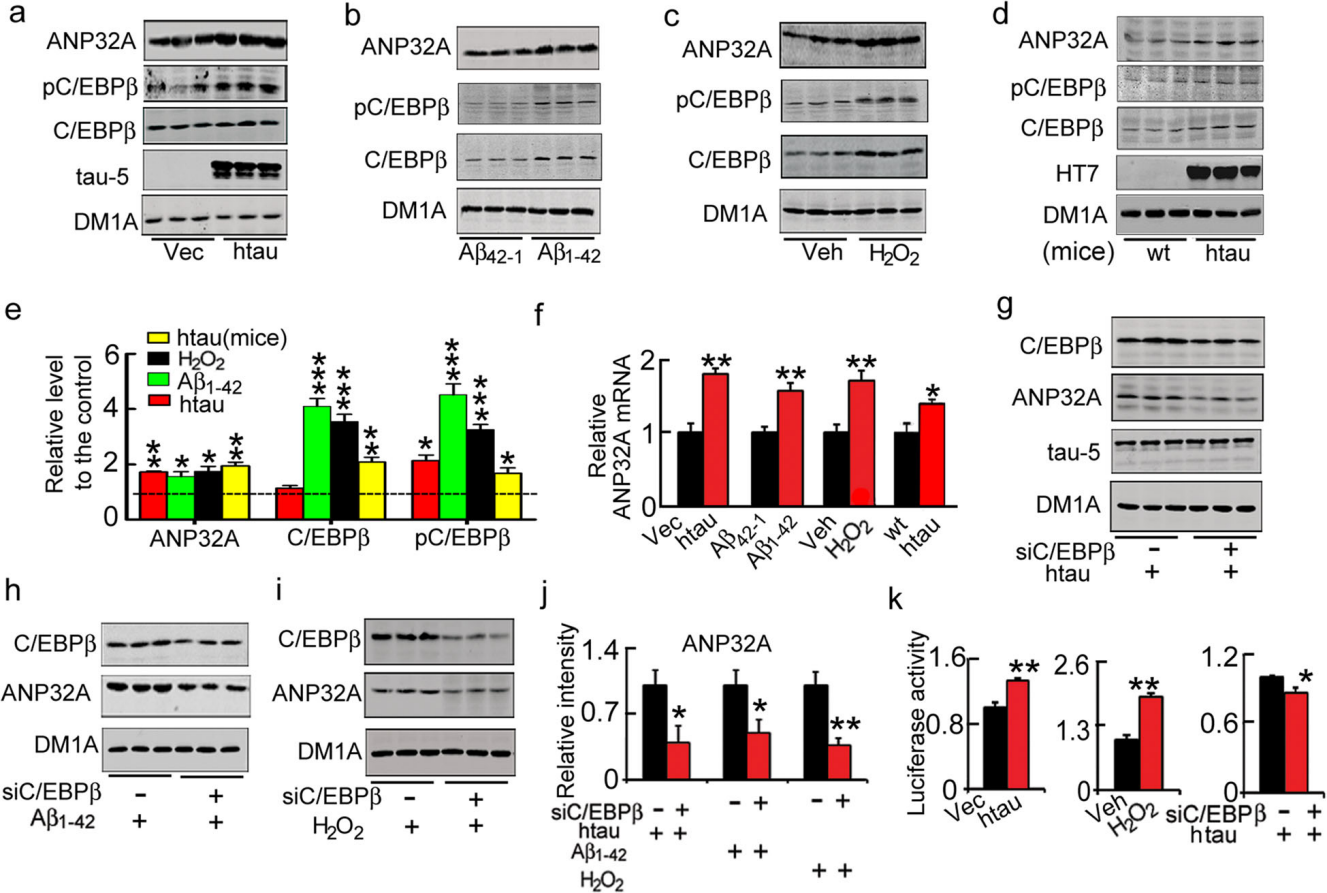
AD-risk factors increase ANP32A *via* activating C/EBPβ. **a** Overexpressing htau in HEK293 cells increased protein level of ANP32A with elevation of total and the phosphorylated transcription factor C/EBPβ (pC/EBPβ) measured by Western blotting, the empty vector (Vec) was transfected as control. **b**, **c** Treatment of HEK293 cells with Aβ_1-42_ (5 μM, 24 h) or H_2_O_2_ (50 μM, 40 min) increased protein level of ANP32A with elevation of total and the pC/EBPβ, and the scrambled Aβ_42-1_ (b) or sterile water (veh, vehicle) (c) was used as control. **d** ANP32A with C/EBPβ and pC/EBPβ levels increased in the hippocampus of 12-month old htau mice compared with the age-matched wild-type (wt) littermates. **e** The quantitative analysis of ANP32A, C/EBPβ and pC/EBPβ level in a-d. The broken line indicates the control level received by the empty vector, Aβ_42-1_, wt or the vehicle. ‘—‘ presents the relative level of control (Vec, Aβ_42-1_, wt or the vehicle). **f** Overexpression of htau and treatment with Aβ_1-42_ (5 μM, 24 h) or H_2_O_2_ (50 μM, 40 min) increased mRNA level of ANP32A in HEK293 cells, and ANP32A mRNA level also increased in the hippocampus of 12-m old htau mice, detected by RT-PCR. **g**–**j** Simultaneous downregulation of C/EBPβ by expressing siC/EBPβ for 48 h attenuated the htau–, Aβ_1-42_–and H_2_O_2_–induced elevation of ANP32A protein in HEK293 cells, detected by Western blotting and quantitative analysis. **k** Overexpression of htau or H_2_O_2_ treatment increased luciferase activity of ANP32A in HEK293 cells, while simultaneous knockdown of C/EBPβ reduced the luciferase activity. Data were presented as mean ± SD. *, *p*<0.05, **, *p*<0.01 *vs* Vec, Aβ_42-1_, wt or the vehicle (Unpaired Student's *t*-test (two-tailed))

To gain insight into the mechanisms underlying the increased ANP32A in AD, HEK293 cells were transiently transfected with pIRES-eGFP-Tau40 or its vector pIRES-eGFP plasmid (Fig. 7a), treated with Aβ oligomers (Fig. 7b) or hydrogen peroxide (H_2_O_2_) (Fig. 7c), the recognized precipitating factors of AD [19, 20]. We observed that htau accumulation, Aβ_1-42_ or H_2_O_2_ treatments induced significant increases of ANP32A protein (Fig. 7a–c,e) and mRNA (Fig. 7f). ANP32A protein and mRNA levels also increased in the hippocampus of 12-month old htau transgenic mice compared with the age-matched wild-type littermates (Fig. 7d–f).

The increase of ANP32A mRNA suggests involvement of the transcription, therefore, we screened for potential binding sites of the ANP32A promoter in transcription factor binding databases [21], and found a well-conserved element for the CCAAT/enhancer binding protein-β (C/EBPβ) in the proximal promoter region of ANP32A. After HEK293 cells transiently transfected with pIRES-eGFP-Tau40, treated with Aβ oligomers or H_2_O_2_, levels of total C/EBPβ and the phosphorylated C/EBPβ at Thr238/188 (pC/EBPβ, the activated site) were found significantly increase (Fig. 7a–c), and total C/EBPβ and the phosphorylated C/EBPβ also increased in 12-m htau mice (Fig. 7d), while knockdown C/EBPβ attenuated the increase of ANP32A (Fig. 7g–j). We also found that htau overexpression by transfection with pIRES-eGFP-Tau40 plasmid or H_2_O_2_ exposure significantly increased the luciferase activity of ANP32A promoter, and siC/EBPβ attenuated the increased luciferase activity of ANP32A induced by htau overexpression (Fig. 7k). These data indicate that AD-related toxic stimuli can increase ANP32A gene transcription with the mechanisms involving C/EBPβ activation.

### Knockdown ANP32A reverses tau phosphorylation by activating PP2A

ANP32A is an endogenous inhibitor of PP2A [22], while PP2A inhibition leading to tau hyperphosphorylation plays an important role in AD neurodegeneration [23]. Therefore, we further measured PP2A activity and tau phosphorylation levels in htau mice. We observed that PP2A activity was decreased and ANP32A knockdown restored PP2A activity (Additional file 3: Figure S1a). Simultaneously, the phosphorylation level of tau at multiple AD-associated sites was significantly increased in htau mice, while silencing ANP32A attenuated tau phosphorylation at Ser231/262/404 and increased unphosphorylated tau level at Ser195/198/199/202 (tau-1) (Additional file 3: Figure S1b, c). These data suggest that the PP2A-assocaited tau dephosphorylation may also contribute to the siANP32A-improved learning and memory in htau mice.

## Discussion

**Fig. 8:**
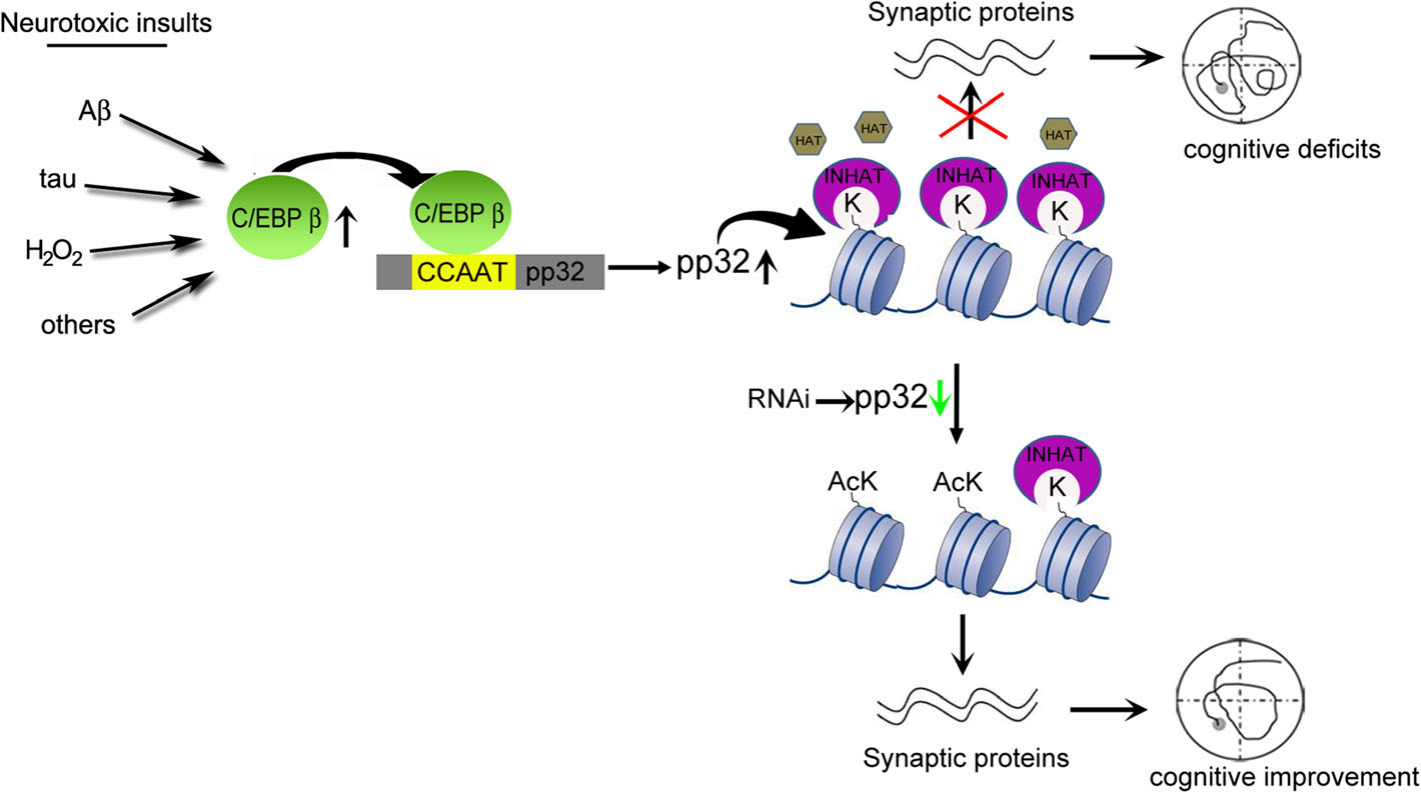
The proposed working model by which ANP32A regulates synaptic plasticity and cognitive function. The neurotoxic insults, such as htau accumulation, or Aβ-fibrils or H_2_O_2_ overproduction, activate C/EBPβ by increasing its protein level and promoting its phosphorylation at Thr238/188. The activated C/EBPβ binds to its responsive element (CCAAT box) in the proximal ANP32A promoter region, and thus stimulates the expression of ANP32A. Then ANP32A interacts with SET to form INHAT, which masks histones for acetylation by HAT. Hypo-acetylation of histone inhibits transcription of synapse-associated proteins, leading to cognitive deficits. Arresting ANP32A overproduction unmasks histone for a robust acetylation, and thus increases the transcription of synaptic plasticity-related proteins to preserve learning and memory capacities

ANP32A is significantly increased in the AD brains [8]. Here we found that protein level of ANP32A was inversely correlated with spatial learning ability in AD mice, knockdown ANP32A rescues learning and memory with preservation of synaptic plasticity. As a subunit of INHAT, ANP32A knockdown can decrease formation of INHAT and thus unmask histone for acetylation that lead to an increased expression of synaptic proteins. The increased synaptic proteins can remodel the synaptic plasticity and ameliorate cognitive deficits (Fig. 8). These findings reveal that ANP32A plays a critical role in learning and memory, and arresting ANP32A elevation in hippocampus may represent a promising therapeutic approach for preserving the cognitive capacity in AD.

The impairment of histone acetylation is causally linked to the cognitive decline in AD [4, 24]. In addition to HATs and HDACs that respectively regulate histone acetylation and deacetylation, INHAT, a complex composed of three essential subunits, TAF-Iα, SET/TAF-Iβ and ANP32A, also affects the acetylation level of histone [5]. INHAT regulates histone acetylation by binding to histones thereby preventing their access to HATs [5]. ANP32A, a key subunit of INHAT, can regulate histone acetylation [6] or the expression of neurofilament light chain by modulating histone acetylation [25], and histone 3 (H3) and H4 acetylation regulates expression of synaptic protein [18]. Here, we show for the first time that ANP32A negatively regulates the expression of a set of synaptic proteins including glutamate receptor subunits GluR1, GluR2, NR2A and NR2B, and as well as Syp and Syn1 *via* suppressing histone acetylation and ANP32A elevation is associated with cognitive impairment in an AD model. Several studies have demonstrated that, genes related to synaptic plasticity such as GluR1, GluR2, NR2A and NR2B, and Syp and Syn1 are downregulated in human AD brains [26–30]. Our results indicate that suppression of histone acetylation induced by the upregulated ANP32A at least contributes to the cognitive decline in AD.

Histone binding is a prerequisite for ANP32A to inhibit HAT activity. Recent study found that ANP32A INHAT domains, a major HAT inhibitory domain of ANP32A residing between amino acids 151 and 180, are responsible for histone binding, HAT inhibitory activity, and repression of transcription. The relative IC_50_ values of the synthesized ANP32A peptide representing ANP32A INHAT domain toward the individual histones H2B, H2A, H3, and H4 ranged between 0.6 and 1.5 μM. Although isolated ANP32A has a high affinity to bind to and inhibit acetylation of histone H2B and H3, this H2B preference is lost when ANP32A is incorporated into the INHAT complex, which predominantly binds to and inhibits acetylation of histones H3 and H4 [5, 6]. Based on the above reasons, we measured the interaction between ANP32A and H3 or H4. We found that knockdown ANP32A decreased the binding protein level with H3 or H4, and increased the acetylated levels of H3K9, H3K14, H4K5 and H4K12. We also found that knockdown ANP32A decreased the interaction of ANP32A with SET and reduced the binding levels of SET with H3 and H4. These findings suggest that knockdown ANP32A not only inhibits INHAT complex formation, but also decreases the interaction of INHAT with histone.

We also identified in the present study that the expression of ANP32A is regulated by C/EBPβ *via* binding to a well-conserved recognition element in the proximal promoter region of ANP32A. C/EBPβ is a transcription factor that belongs to the C/EBP family, which plays a central role in cell differentiation and cell lineage definition, as well as in inflammation control [31]. Of note, C/EBPβ is increased in the brains of AD patients and the AD transgenic animal models [32, 33]. It is conceivable that the upregulation of ANP32A in AD may be a result of the increased C/EBPβ. Interestingly, we find that different type of AD-associated stressors (e.g. tau overexpression, Aβ exposure, and oxidative stress) can increase the levels of total and the phosphorylated C/EBPβ. This result indicates that upregulation of C/EBPβ may represent a common pathway leading to overexpression of ANP32A, followed by impairment of histone acetylation and cognitive deficits.

## Conclusion

Taken together, our study establishes that arresting ANP32A elevation in AD mice rescues the cognitive functions by unmasking histone for a robust acetylation and thus increasing expression of synapse-associated proteins with reconstruction of synapse morphology and the functions.

## Additional files

Additional file 1: Table S1. Antibodies employed in the study. (DOC 61 kb)

Additional file 2: The supplementary materials and methods used in this study. (DOC 40 kb)

Additional file 3: Figure S1. Downregulating ANP32A ameliorates tau phosphorylation in htau mice. (DOC 245 kb)

## Abbreviations

AD: Alzheimer’s disease
C/EBPβ: CCAAT/enhancer binding protein-β
HATs: Histone acetyltransferases
HDACs: Histone deacetylases
htau mice: Human tau transgenic mice
INHAT: Inhibitor of acetyltransferases
MWM: Morris water maze
NR2A: N-methyl-D-aspartate receptor type 2A
NR2B: N-methyl-D-aspartate receptor type 2B
Syp: Synaptophysin
Syn1: Synapsin I
