# Supplementary material for: Downregulating ANP32A rescues synapse and memory loss via chromatin remodeling in Alzheimer model
Source: Mol Neurodegener. 2017 May 4;12:34. doi: 10.1186/s13024-017-0178-8 (PMC5418850; doi:10.1186/s13024-017-0178-8)
Supplement: Supplementary file 1 — Antibodies employed in the study. (DOC 61 kb) [file 13024_2017_178_MOESM1_ESM.doc]

Table S1. Antibodies employed in the study.

| Antibody | Specific | type | WB | IF | Source |
| --- | --- | --- | --- | --- | --- |
| ANP32A | PHAP1 | Poly- | 1:1000 | 1:200 | Abcam |
| H3K14 | Histone H3 acetylated at lysine 14 | Poly- | 1:500 |  | Cell Signaling |
| H3K9 | Histone H3 acetylated at lysine 9 | Poly- | 1:1000 | 1:200 | Abcam |
| H4K8 | Histone H4 acetylated at lysine 8 | Poly- | 1:500 | 1:200 | Millipore |
| H4K12 | Histone H4 acetylated at lysine 12 | Poly- | 1:500 | 1:200 | Abcam |
| Histone H4 | C-terminus of Human Histone H4 | Poly- | 1:1000 |  | Abcam |
| Histone H3 | C-terminus of Human Histone H3 | Poly- | 1:1000 |  | Abcam |
| DM1A | Total β-tubulin | Mono- | 1:1000 |  | Sigma |
| β-Actin | Total β-actin | Mono- | 1:1000 |  | Abcam |
| pS262 | P-tau at Ser262 | Poly- | 1:1000 |  | Signalway Antibody |
| pT231 | P-tau at Thr231 | Poly- | 1:1000 |  | Signalway Antibody |
| pS404 | P-tau at Ser404 | Poly- | 1:1000 |  | Signalway Antibody |
| tau-1 | DeP-tau at Ser195/198/199/202 | Mono- | 1:1000 |  | Millipore |
| AT8 | P-human tau at Ser202/205 | Mono- | 1:1000 |  | Thermo Sci Pierce |
| tau-5 | Total tau | Mono- | 1:1000 |  | Millipore |
| NR2A | NMDAR2A C-term | Poly- | 1:1000 |  | Millipore |
| NR2B | NMDAR2B C-term | Poly- | 1:1000 |  | Abcam |
| SYN1 | Synapsin-1 C-term | Poly- | 1:1000 |  | Millipore |
| SYP | Total Synaptophysin | Mono- | 1:1000 |  | Millipore |
| C/EBP β | Total C/EBP β | Poly- | 1:1000 |  | Abcam |
| pC/EBP β | P-C/EBP β at Thr235/188 | Poly- | 1:500 |  | Abcam |
| SET | Total I2PP2A | Mono- | 1:1000 |  | Santa Cruz |
| GluR1 | Total GluR1 | Poly- | 1:1000 |  | Millipore |
| GluR2 | Total GluR2 | Mono- | 1:500 |  | Millipore |

Note：’P’ means phosphorylated; ‘DeP’ means dephosphorylated.
