# Supplementary material for: Downregulating ANP32A rescues synapse and memory loss via chromatin remodeling in Alzheimer model
Source: Mol Neurodegener. 2017 May 4;12:34. doi: 10.1186/s13024-017-0178-8 (PMC5418850; doi:10.1186/s13024-017-0178-8)
Supplement: Supplementary file 2 — The supplementary materials and methods used in this study. (DOC 40 kb) [file 13024_2017_178_MOESM2_ESM.doc]

**
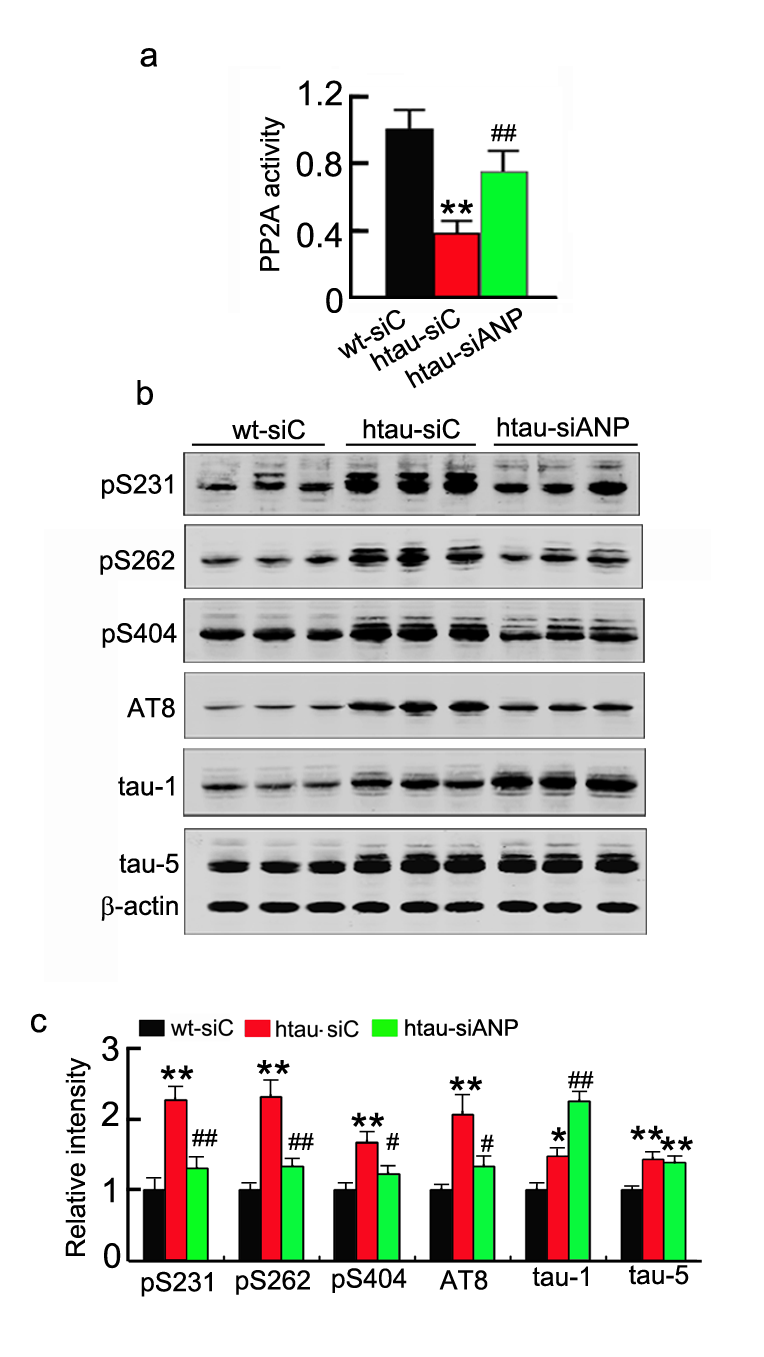
**

**Additiional file 2: Figure S1 Downregulating ANP32A ameliorates tau phosphorylation in htau mice.**

(a) The activity was decreased in hippocampal CA3 extracts of htau mice, while siANP32A reactivated PP2A, measured by using a Serine/Threonine Phosphatase Assay Kit (n = 3-5 in triplicates).

(b, c) Downregulating ANP32A decreased tau phosphorylation at multiple AD-associated sites, detected by Western blotting.

Data were presented as mean±SD. *, *p*<0.05, **, *p*<0.01 *vs* wt-siC; #, *p*<0.05; ##, *p*<0.01 *vs* htau-siC (two-way ANOVA followed by Bonferroni’ s post hoc test).
