# Supplementary material for: Downregulating ANP32A rescues synapse and memory loss via chromatin remodeling in Alzheimer model
Source: Mol Neurodegener. 2017 May 4;12:34. doi: 10.1186/s13024-017-0178-8 (PMC5418850; doi:10.1186/s13024-017-0178-8)
Supplement: Supplementary file 3 — Downregulating ANP32A ameliorates tau phosphorylation in htau mice. (DOC 245 kb) [file 13024_2017_178_MOESM3_ESM.doc]

# Downregulating ANP32A rescues synapse and memory loss via chromatin remodeling in Alzheimer model

Gao-Shang Chai, Qiong Feng, Zhi-Hao Wang, Yu Hu, Dong-Sheng Sun, Xiao-Guang Li, Dan Kea, Hong-lian Li, Gong-Ping Liu, Jian-Zhi Wang

**Methods**

**Plasmids**

For measuring the transcription activity, the plasmids containing ANP32A gene promoters were fused to the luciferase reporter gene of pGL3-basic purchased from Promega (Madison, WI). The primers for ANP32A were 5’-GCGTGCTAGCCCGGGCTCGAGTGTTTTATTTACATTGTCACT-3’ and 5’-AACAGTACCGGAATGCCAAGCTTGAGGCTCCCGCGCCGGCGGAATT-3’ according to human HEK293 genomic DNA. The PCR product was digested by BglII and HindIII and ligated into pGL3-basic, thereby generating ANP32A-Luc plasmid. Human pIRES-eGFP-Tau40 (the longest human tau which containing 4-repeat isoform with a total size of 441 amino acids) and its vector pIRES-eGFP plasmids were gifts of Dr. Khalid Iqbal (New York State Institute for Basic Research in Developmental Disabilities, Staten Island, NY). The siC/EBPβ plasmid was the gifted from Prof. Keqiang Ye (Department of Pathology and Laboratory Medicine, Emory University School of Medicine, Atlanta, GA).

**Cell culture, transfection and reporter gene assays**

The HEK293 cells were cultured in DMEM supplemented with 10% fetal bovine serum (GIBCO), 100 U/ml penicillin and 100 mg/ml streptomycin (Invitrogen) in a humidified atmosphere of 5% CO_2_ in air at 37 °C. The cells were plated onto six-well plates overnight, and the plasmids were transfected the next day using Lipofectamine 2000 according to the manufacturer’s instruction (Invitrogen, CA).

To assay the luciferase activity, HEK293 cells were seeded into 24-well plates in DMEM, one day prior to transfection, and co-transfected with pGL3-construct (ANP32A-Luc) and pRL-TK with pIRES-eGFP-Tau40 or the cells treatment with Aβ oligomers or H_2_O_2_. And then cells were harvested and lysed with 100 μl Passive Lysis Buffer. The cell extracts (20 μl) were used for luciferase activity assay using a Lumat LB9507 luminometer (Berthold) and the Dual Luciferase Reporter (DLR) assay system (Promega), by following the supplier’s instruction.

**Aβ _(1-42)_ peptide preparation**

Aβ_1-42_ peptide and reverse Aβ_42-1_ peptide were supplied by Sigma (St Louis, MO, USA, A9810, SCP0048) and oligomerized by the procedure described previously [34]. Briefly, prior to use, The Aβ_1-42_ or Aβ_42-1_ peptide was dissolved in dimethyl sulfoxide and diluted in sterile and commercial physiological saline. Then, the solution was incubated for oligomerization at 37 °C in dark for 1 week before use.

**PP2A activity assay**

PP2A activity was measured using a Serine/Threonine Phosphatase Assay Kit by following the manufacturer's instructions, which can specifically measure PP2A but not the other protein phosphatases through selection of specific substrate and alternative buffer systems (Promega, MA). The absorbance was read at 600 nm (BioTek Instruments, VT).

**References:**

34. Zeng J, Jiang X, Hu XF, Ma RH, Chai GS, Sun DS, Xu ZP, Li L, Bao J, Feng Q, Hu Y, Chu J, Chai DM, Hong XY, Wang JZ, Liu GP. Spatial training promotes short-term survival and neuron-like differentiation of newborn cells in Aβ1-42-injected rats. Neurobiol Aging. 2016;45:64-75.
